# A Biological Hierarchical Model Based Underwater Moving Object Detection

**DOI:** 10.1155/2014/609801

**Published:** 2014-07-22

**Authors:** Jie Shen, Tanghuai Fan, Min Tang, Qian Zhang, Zhen Sun, Fengchen Huang

**Affiliations:** ^1^College of Computer and Information, Hohai University, Nanjing 210098, China; ^2^College of Communication Engineering, PLA University of Science and Technology, Nanjing 210007, China; ^3^School of Information Engineering, Nanchang Institute of Technology, Nanchang 330099, China

## Abstract

Underwater moving object detection is the key for many underwater computer vision tasks, such as object recognizing, locating, and tracking. Considering the super ability in visual sensing of the underwater habitats, the visual mechanism of aquatic animals is generally regarded as the cue for establishing bionic models which are more adaptive to the underwater environments. However, the low accuracy rate and the absence of the prior knowledge learning limit their adaptation in underwater applications. Aiming to solve the problems originated from the inhomogeneous lumination and the unstable background, the mechanism of the visual information sensing and processing pattern from the eye of frogs are imitated to produce a hierarchical background model for detecting underwater objects. Firstly, the image is segmented into several subblocks. The intensity information is extracted for establishing background model which could roughly identify the object and the background regions. The texture feature of each pixel in the rough object region is further analyzed to generate the object contour precisely. Experimental results demonstrate that the proposed method gives a better performance. Compared to the traditional Gaussian background model, the completeness of the object detection is 97.92% with only 0.94% of the background region that is included in the detection results.

## 1. Introduction

Underwater object detection is aiming to extract the interesting objects from the background scene. Effective underwater moving object detection contributes to many scientific research and engineering applications, such as marine biology, seabed topography, marine environment monitoring, and marine exploration [1]. However, due to the strong optical attenuation and light scattering caused by the water medium and suspending particles, underwater images are essentially characterized by their poor visibility, especially the low contrast and distorted information [2, 3]. These low quality image data seriously block the underwater computer vision tasks. In the underwater object detection task, the decayed color and the haze effect would significantly decrease the contrast between the object and the background. Many commonly used image features are distorted and can hardly be taken for precise object detection.

After a long period of evolution, biological visual systems develop a strong ability for sensing the world. Various visual mechanisms in animals have been simulated and introduced into computer vision tasks [4–6]. For underwater object detection, the visual system in aquatic animals gives us many valuable inspirations and some progress has been achieved in the bionic model. Barat and Rendas [7] introduced the motion information in successive video frames to extract salient regions. The edge and contour of the object are detected by the active contour algorithm. Walther et al. [8] combined the visual attention model and the background difference to obtain global saliency maps. Wang et al. [9] updated the Itti model by introducing the prior knowledge about the maximum number of objects in a single frame. However, many problems still exist in these researches. The underwater object detection by the above methods is incomplete, missing in object regions. Furthermore models based on the prior knowledge learning are difficult to adapt for the underwater tasks. Most crucially the artificial illumination which is used to compensate for the power attenuation in the underwater medium would generate inhomogeneous illumination environments while the background scene is unstable and the pixels with strong intensity would be mistaken as the object region.

In order to solve the problems in existing underwater moving object detection method, this paper proposes a novel hierarchical background model by simulating the frog visual perception, which is considered to have an excellent ability for motion detection [10]. Hence the visual relativity is modeled. Only the intensity information is extracted and introduced into the background model. Finally a hierarchical background modeling is proposed for efficiently detecting the underwater object in illumination changing and nonstationary environments. With the bionic method, the proposed method has high dynamical adaptability in the underwater object extraction task and stronger robustness to the underwater environment. The experiment results prove its efficiency in object extraction under the underwater optical environments.

The remainder of this paper is organized as follows.  Section 2 briefly describes the characteristics of visual perception and information processing mode in the frogs.  Section 3 presents details of the proposed method. The experiment results on several underwater image sequences are discussed in Section 4 and finally Section 5 concludes the paper.

## 2. Frog Visual Mechanism and Information Processing Model

Frog is a typical visually guided animal. The eye of the frogs is their main biological sensor for tracking preys. However frogs are more sensitive to the moving object compared with other animals. When frogs keep completely static, nothing can be perceived by the retina of the eye. Therefore they are blind to the static object even if it is very close [11]. Accordingly the motion information of moving object is the critical cue which controls the preying behavior in the visual system of frogs. Biological researches find that frogs are born “myopia.” The foreground scene is clearly imaged on the retina, while the background is blurred. This visual mechanism enables frogs to find and capture the preys correctly and quickly. Different from the focus shift process in human visual attention mechanism [12], frogs keeping in static state do not move their eyes to search and track interested objects. However if the frog's body moves, the whole visual scene would be reversal [13]. In this case, in order to keep the image stably represented on the retina, the frogs would move the eye to compensate for the movement of the scene.

In the view of the underwater moving object detection, this paper focuses on three aspects in frog's visual and neurophysiologic mechanism.

1. The low distance resolution would result in the blurred background and clear foreground presented in the retina. Therefore frog can easily distinguish the object in the foreground from the background. By employing this mechanism in the computer vision, we firstly segment the image into subblocks which are utilized for classification of the foreground and the background. Then the background region is ignored and the pixel-based processing is operated on the foreground region. With the above preprocessing, the foreground region can be easily extracted and the object detection operation is focused on the foreground region. Accordingly the redundant computation on the background region is saved and the complexity of the object detection is reduced. Furthermore the subblocks based processing solves the difficulties caused by nonstationary background in some extent.
2. A frog has a memory on both the moving objects and the background. Once the interest is focused on any objects, the attention of frogs can hardly be dispersed. By taking this into consideration, the foreground and the background are modelled and updated by the feature extracted in the respective regions. This strategy solves the problems caused by the change in the lamination and increases the accuracy of underwater object detection.
3. The retina and neural fiber in the eye of frogs are sensitive to the local bright-dark contrast and the bright and dark change in movement region. This visual sensitivity can be modeled by the selection of the image feature. According to the computer vision task, the intensity and the texture feature describing the intensity distribution in local regions are extracted for detecting the contour of the moving object.

Inspired by the above aspects of visual mechanisms in the eye of frogs, this paper proposed a hierarchical background model based underwater moving object detection method. In this method, the foreground and the background are modeled by the information extracted from pixels and subblocks, respectively. The intensity and the texture feature are extracted to describe the contour of the underwater objects correctly.

## 3. Object Detection Method

### 3.1. Overview of the Proposed Method

The key for the object extraction is to stretch the contrast between the object and the background. Considering the spatial correlation between pixels, the subblock based background modeling is sensitive to the global change of the scene but blind to local movement which solves the problems caused by the unstable background. However it might generate the rough object region with serious blocking effect due to subblock operation which may deform the object and the intensity feature for modeling the background can hardly identify the objects in the scene in some cases.

More precise object information can be extracted by using pixel-based background model. By using the pixel-based operation, the rough object region is correctly detected without the blocking effect. However, the results given by the pixel-based operation do not only include the object region but also include the regions surrounding the object. Hence, errors would exist in the scene with the unstable background.

**Figure 1:**
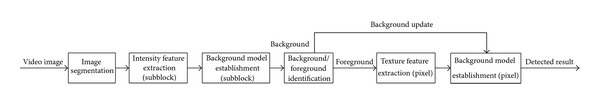
Block diagram of the proposed underwater moving object detection method.

Therefore, the subblock and the pixel-based operation are mutually compensative. The asymmetric forward feedback mechanism is then applied to jointly combine these two strategies to form a hierarchical background model for object detection. Firstly, intensity features are extracted in the subblock and the difference between the subblocks is taken as the cue for classifying the rough object and background region. The rough object region is extracted afterwards and the background model is updated. Then texture features of every single pixel which belongs to the rough object region are extracted to establish the pixel-based background model.  Figure 1 illustrates the process of the proposed underwater moving object detection algorithm.

In order to reduce the computational complexity, the detection process is operated under the following rules.

1. The background region identified by the subblock based method is reliable. The pixel-based identification is omitted for the given background region. In order to adapt our method to the change of the scene, the background region is updated by the information extracted from the subblock regions but not the pixels.
2. The foreground region identified by the subblock based method contains the pixels of the real object region and a small amount of unstable pixels. Hence, the pixel-based algorithm should be utilized to further detect the object region to remove the blocking effect.
3. Since most of the pixels in the detected rough object region are included in the real object region, updating process of the background model is not necessary in this region.

### 3.2. Rough Object Region Detection

The rough object region is detected by the subblock based operation. The input video frames are segmented into multiple nonoverlapped subblocks with a size of *M* × *N*. For each subblock, the intensity feature is extracted. By block truncation coding (BTC), an image coding method which represents the movement vector based on the subblock [14], the intensity feature vector accordingly can be represented as *v* = {*μ*_*ht*_, *μ*_*hb*_, *μ*_*lt*_, *μ*_*lb*_}:  

(1) $$\begin{array}{c} \mu =\frac{1}{M\times N}\overset{M}{\underset{m=1}{\sum }}\overset{N}{\underset{n=1}{\sum }}x_{m,n},\\ r\mu_{h}=\frac{\sum_{m=1}^{M}\sum_{n=1}^{N}\left(x_{m,n}\;|\;x_{m,n}\geq \mu \right)}{\sum_{m=1}^{M}\sum_{n=1}^{N}\{\begin{array}{c} 1,\;\;x_{m,n}\geq \mu \\ 0,\;\;\text{others}\;\; \end{array}},\\ \mu_{l}=\frac{\sum_{m=1}^{M}\sum_{n=1}^{N}\left(x_{m,n}\;|\;x_{m,n}<\mu \right)}{\sum_{m=1}^{M}\sum_{n=1}^{N}\{\begin{array}{c} 1,\;\;x_{m,n}<\mu \\ 0,\;\;\text{others}\;\; \end{array}},\\ \mu_{ht}=\frac{\sum_{m=1}^{M}\sum_{n=1}^{N}\left(x_{m,n}\;|\;x_{m,n}\geq \mu_{h}\right)}{\sum_{m=1}^{M}\sum_{n=1}^{N}\{\begin{array}{c} 1,\;\;x_{m,n}\geq \mu_{h}\\ 0,\;\;\text{others}\;\;\; \end{array}},\\ \mu_{hb}=\frac{\sum_{m=1}^{M}\sum_{n=1}^{N}\left(x_{m,n}\;|\;\mu \leq x_{m,n}<\mu_{h}\right)}{\sum_{m=1}^{M}\sum_{n=1}^{N}\{\begin{array}{c} 1,\;\;\mu \leq x_{m,n}<\mu_{h}\\ 0,\;\;\text{others}\;\;\;\; \end{array}},\\ \mu_{lt}=\frac{\sum_{m=1}^{M}\sum_{n=1}^{N}\left(x_{m,n}\;|\;\mu_{l}\leq x_{m,n}<\mu \right)}{\sum_{m=1}^{M}\sum_{n=1}^{N}\{\begin{array}{c} 1,\;\;\mu_{l}\leq x_{m,n}<\mu \\ 0,\;\;\text{others}\;\;\;\;\;\;\; \end{array}},\\ \mu_{lb}=\frac{\sum_{m=1}^{M}\sum_{n=1}^{N}\left(x_{m,n}\;|\;x_{m,n}<\mu_{l}\right)}{\sum_{m=1}^{M}\sum_{n=1}^{N}\{\begin{array}{c} 1,\;\;x_{m,n}<\mu_{l}\\ 0,\;\;\text{others}\;\; \end{array}}, \end{array}$$

where *x*_*m*,*n*_ denotes the intensity of pixel (*m*, *n*) in a subblock, *μ* denotes the mean intensity of all pixels in a subblock, and *μ*_*h*_ is the mean intensity of the pixels whose intensity is higher than the threshold *μ* while *μ*_*l*_ denotes the mean intensity of the pixels whose intensity is lower than the value of *μ*.

The feature extracted in a subblock is represented by a vector *ν* = {*μ*_*ht*_, *μ*_*hb*_, *μ*_*lt*_, *μ*_*lb*_}. If all *x*_*m*,*n*_ in one subblock are identical, then set all these four values as *μ*. If the high-intensity values of pixels in one subblock are identical, then set *μ*_*ht*_ and *μ*_*hb*_ as *μ*_*h*_. If the low-intensity values of pixels in one subblock are identical, then set *μ*_*lt*_ and *μ*_*lb*_ as *μ*_*l*_. With the subblock based background modeling method and the intensity feature, the difference between the object and the background region can be correctly and quickly recognized. In order to solve the problem caused by the change of the scene, the strategy for Gaussian mixture model updating [15] is introduced.

A set of intensity feature vectors {*v*_0_^*i*^, *v*_1_^*i*^,…, *v*_*K*−1_^*i*^} for each subblock *i* is introduced. In order to indicate the importance of different elements, the additional weight *ω*_*k*_^*i*^ is introduced and ∑_*k*_ *ω*_*k*_^*i*^ = 1. Accordingly, the vector with larger weights has stronger ability to identify the object and the background. These weights are initialized as

(2) $$\begin{array}{c} v_{0}^{i}=v^{i},\\ \omega_{0}^{i}=1,\\ v_{1}^{i}=v_{2}^{i}=\cdots =v_{K-1}^{i}=0,\\ \omega_{1}^{i}=\omega_{2}^{i}=\cdots =\omega_{K-1}^{i}=0, \end{array}$$

where *ν* ^*i*^ denotes the vector of the intensity feature extracted from subblock *i* in the first frame. Each subblock in the following frame is discriminated according to

(3) $$\begin{array}{c} B_{i}=\arg\;\min\left(\overset{b}{\underset{k=1}{\sum }}\omega_{k,t}^{i}>T\right), \end{array}$$

where *w*_*k*,*t*_^*i*^ is the weight for the *t*th frame and the threshold *T* is set for identifying the rough region of the object and the background. The first *B*_*i*_ vectors which are satisfied with (3) are discriminated as the background regions, and the last *K* − *B*_*i*_ vectors belong to the object regions.

To extract the rough object region, the intensity features in the subblocks are extracted. Then they are related to the background model by the Euclidean distance:

(4) $$\begin{array}{c} D\left(v_{t}^{i},v_{k,t}^{i}\right)=\sqrt{\overset{4}{\underset{j=1}{\sum }}{\left(v_{tj}^{i}-v_{k,tj}^{i}\right)}^{2}}, \end{array}$$

where *v*_*tj*_^*i*^ and *v*_*k*,*tj*_^*i*^ are the *j*th vector of *v*_*t*_^*i*^ and *v*_*k*,*t*_^*i*^. If *D*(*v*_*t*_^*i*^, *v*_*k*,*t*_^*i*^) < *T*_*D*_ (*T*_*D*_ is the distance threshold), the intensity feature of subblock *i* and *k*th vector are matched. Once a subblock matches the first *B*_*i*_ vectors, it belongs to the background or it is involved in the object region.

If the feature of subblock *i* is in correspondence with at least one background model, then *v*_*t*_^*i*^ is utilized to update the background model:

(5) $$\begin{array}{c} v_{o,t+1}^{i}=\left(1-\alpha_{b}\right)v_{o,t}^{i}+\alpha_{b}v_{t}^{i}, \end{array}$$

where *α*_*b*_ is the parameter controlling the rate of learning. The parameters of the background model are updated as follows:

(6) $$\begin{array}{c} \omega_{k,t+1}^{i}=\left(1-\alpha_{\omega }\right)\omega_{k,t}^{i}+\alpha_{\omega }M_{K},\\ \omega_{k,t+1}^{i}=\frac{\omega_{k,t+1}^{i}}{\sum_{m=1}^{K}\omega_{m,t+1}^{i}}, \end{array}$$

where *α*_*ω*_ is the parameter controlling the rate of learning; *M*_*K*_ = 1 when the new subblock *i* matches the *k*th vector, and *M*_*K*_ = 0 otherwise.

If subblock *i* fails to match any models, then a new model is established with a minimum weight *v*_*t*_^*i*^. The new model is initialized as

(7) $$\begin{array}{c} \omega_{n,t+1}^{i}=\alpha_{\omega },\\ \nu_{n,t+1}^{i}=\nu_{t}^{i}. \end{array}$$

If the variance of the interest subblock is different from that of the background model, the interest subblock is likely to belong to the moving object region but not the background. To solve this problem and considering the large influence of the illumination on the imaging environments the threshold *T*_*D*_ is adaptively moderated by the intensity variance:

(8) $$\begin{array}{c} T_{D}=T_{D}\left(\alpha +S\right), \end{array}$$

where *α* is an empirical constant and set as 0.7 ≤ *α* ≤ 0.8. The parameter *S* denotes the similarity of intensity features between two subblocks:

(9) $$\begin{array}{c} S=\frac{\left|U_{A}•U_{B}^{T}\right|}{||U_{A}||•||U_{B}||},\\ U_{A}=\left\{\left|\mu_{A}-x_{0}^{A}\right|,\left|\mu_{A}-x_{1}^{A}\right|,\ldots ,\left|\mu_{A}-x_{MN-1}^{A}\right|\right\},\\ U_{B}=\left\{\left|\mu_{B}-x_{0}^{B}\right|,\left|\mu_{B}-x_{1}^{B}\right|,\ldots ,\left|\mu_{B}-x_{MN-1}^{B}\right|\right\}, \end{array}$$

where *μ*_*A*_ and *μ*_*B*_ denote the mean intensity of all pixels in two subblocks, respectively. *x*_*i*_^*A*^ and *x*_*i*_^*B*^ are the intensities of *i*th pixel in two subblocks, respectively.

### 3.3. Accurate Object Contour Extraction

For each pixel in the detected rough object region, the texture feature is extracted and utilized to extract the accurate object contour. In this paper, we choose the local binary pattern (LBP) texture operator to describe texture features. The most important properties of the LBP operator are its tolerance against the change of illumination and its computational simplicity [16–18]. In order to adapt the LBP to the underwater scenes, we modify this operator.

**Figure 2:**
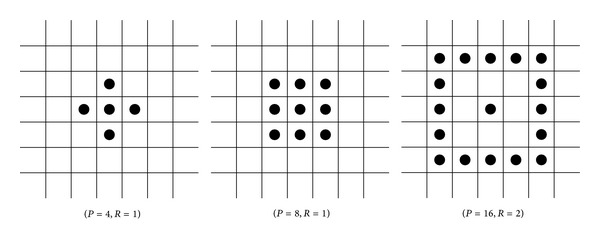
Neighborhood with different *P* and *R*.

Given the center pixel (*x*_*c*_, *y*_*c*_), LBP operator uses joint distribution to describe local texture features:

(10) $$\begin{array}{c} T=t\left(g_{c},g_{0},\ldots ,g_{P-1}\right), \end{array}$$

where *g*_*c*_ corresponds to the gray value of the pixel (*x*_*c*_, *y*_*c*_) and *g*_*p*_  (*p* = 0,1,…, *P* − 1) are the gray values of pixels which are equally located on a circle with radius *R*. By increasing the radius, we can collect larger-scale texture primitives as shown in Figure 2.

By introducing the difference between *g*_*c*_ and *g*_*p*_, the joint distribution *T* can be transformed as

(11) $$\begin{array}{c} T=t\left(g_{c},g_{0}-g_{c},\ldots ,g_{P-1}-g_{c}\right). \end{array}$$

Assuming that *g*_*c*_ and *g*_*p*_ are independent, *T* can be decomposed as

(12) $$\begin{array}{c} T\approx t\left(g_{c}\right)t\left(g_{0}-g_{c},\ldots ,g_{P-1}-g_{c}\right). \end{array}$$

As *t*(*g*_*c*_) denotes the gray distribution of the whole image, the texture feature can be described by the joint distribution of the gray difference between the pixels *P* and the center pixel (*x*_*c*_, *y*_*c*_), as

(13) $$\begin{array}{c} T\approx t\left(g_{0}-g_{c},\ldots ,g_{P-1}-g_{c}\right). \end{array}$$

If illumination changes linearly in underwater scenes, the value of *g*_*p*_ − *g*_*c*_ is not changed. Hence, the sign function can be chosen as the replacement to describe the texture feature:

(14) $$\begin{array}{c} T\approx t\left(s\left(g_{0}-g_{c}\right),s\left(g_{1}-g_{c}\right),\ldots ,s\left(g_{p-1}-g_{c}\right)\right), \end{array}$$

where the sign function can be denoted as

(15) $$\begin{array}{c} s\left(x\right)=\{\begin{array}{cc} 1, & x\geq 0,\\ 0, & x<0. \end{array} \end{array}$$

Practically the sign of the differences in a neighborhood is interpreted as a *P*-bit binary number. This 2^*p*^-bit value is transformed into a unique decimal number for describing the local spatial texture feature:

(16) $$\begin{array}{c} \text{LB}{\text{P}}_{P,R}=\overset{P-1}{\underset{p=0}{\sum }}s\left(g_{p}-g_{c}\right)2^{p}. \end{array}$$

LBP is robust against the considerable gray-scale variations which commonly appear in natural images. Moreover, the LBP operator is computationally economic, which is important in practice. Besides these factors, LBP is a nonparametric method without any assumptions about the underlying distributions. However since the low change of the grey in the underwater background, the grey values between the center point and its neighborhood are homogeneous. In this case, a large error would exist if the traditional LBP operator is used. For example, if *g*_*c*_ = 20 and *g*_*p*_ = 19 the *s*(*x*) given by (15) is 0, while *s*(*x*) = 1 when *g*_*p*_ = 19. In practice, this low difference between *g*_*c*_ and *g*_*p*_ is commonly ignored. Hence a moderation factor *β* is introduced and *s*(*g*_*p*_ − *g*_*c*_) in (16) is replaced by *s*(*g*_*p*_ − *g*_*c*_ + *β*). In this paper, we set *β* = 3.

A set of texture feature vectors {*h*_0_^*i*^, *h*_1_^*i*^, *L*, *h*_*K*−1_^*i*^} is extracted within the rough object region. These features are arranged according to the image sequence. The texture vectors are initialized as

(17) $$\begin{array}{c} h_{0}^{i}=h^{i},\\ h_{1}^{i}=h_{2}^{i}=\cdots =h_{K-1}^{i}=0, \end{array}$$

where *h* ^*i*^ denotes the texture feature of LBP in the pixel *i* of the first frame. Euclidean distance which is to estimate the similarity between *h*_*t*_^*i*^ and {*h*_0_^*i*^, *h*_1_^*i*^,…, *h*_*K*−1_^*i*^} is calculated as

(18) $$\begin{array}{c} D\left(h_{t}^{i},h_{t,j}^{i}\right)=\sqrt{\overset{K-1}{\underset{j=0}{\sum }}{\left(h_{t}^{i}-h_{t,j}^{i}\right)}^{2}}, \end{array}$$

where *j*  (*j* = 1,2,…, *K* − 1) denotes the *j*th component of the texture feature vectors. If *D*(*h* ^*i*^, *h*_*t*,*j*_^*i*^) < *T*_*D*_, pixel *i* is identified as object. Otherwise, pixel *i* is defined as the background and this texture feature is introduced into the model *h*_*t*_^*i*^:

(19) $$\begin{array}{c} h_{t+1,j}^{i}=h_{t,j-1}^{i},\\ h_{t+1,0}^{i}=h_{t}^{i}. \end{array}$$

The hierarchical background modeling method can be summarized as shown in Algorithm 1.

## 4. Experimental Results and Analysis

**Figure 3:**
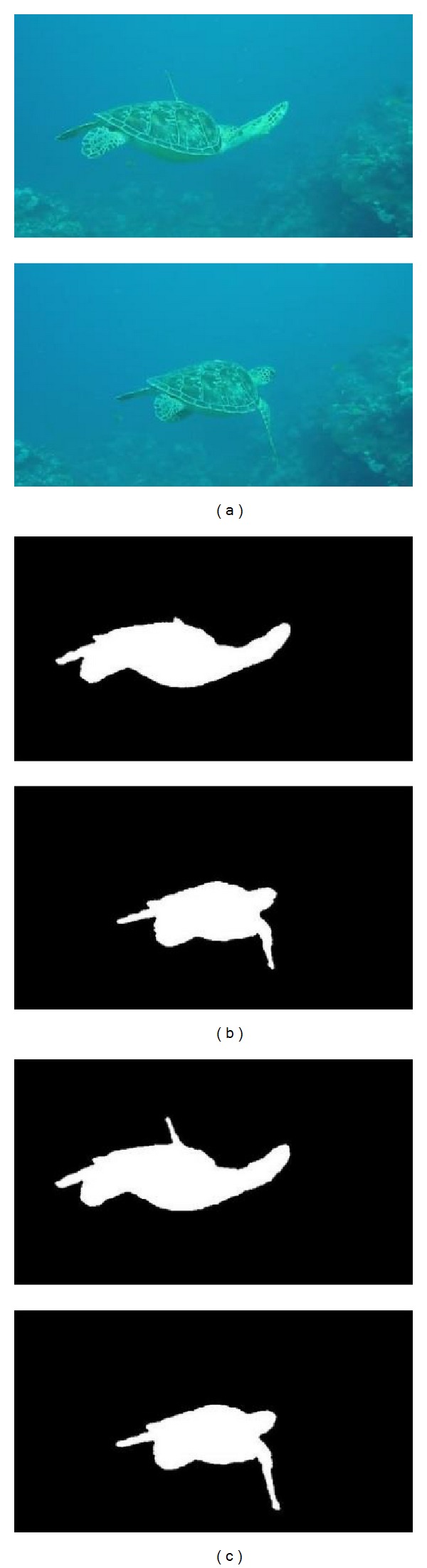
Results of close moving object detection. (a) Original images. (b) Gaussian background modeling method. (c) The proposed method.

**Figure 4:**
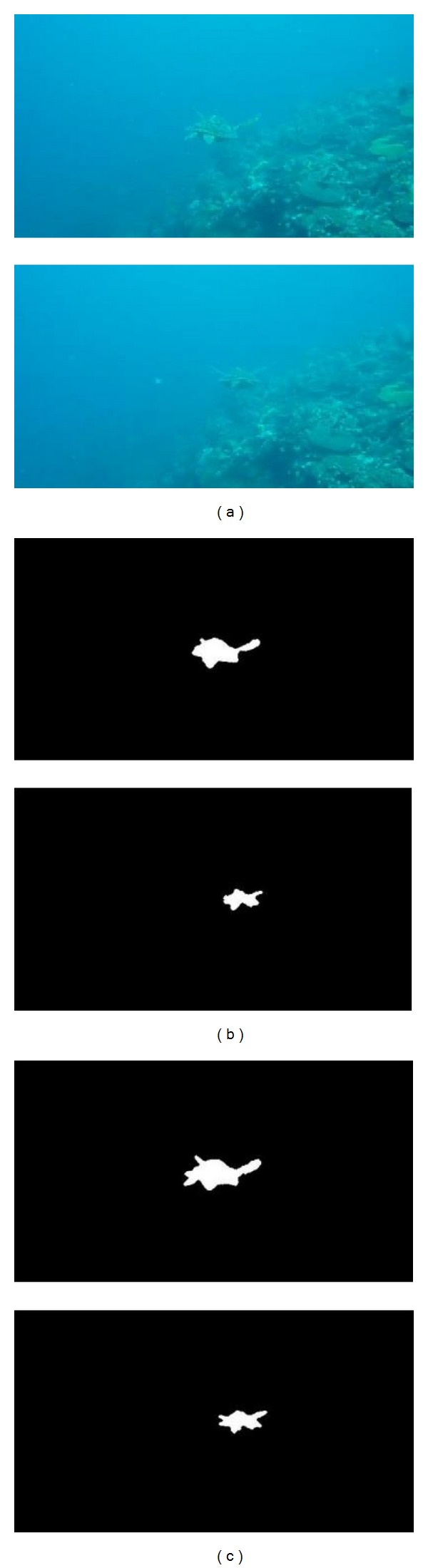
Results of distant moving object detection. (a) Original images. (b) Gaussian background modeling method. (c) The proposed method.

**Figure 5:**
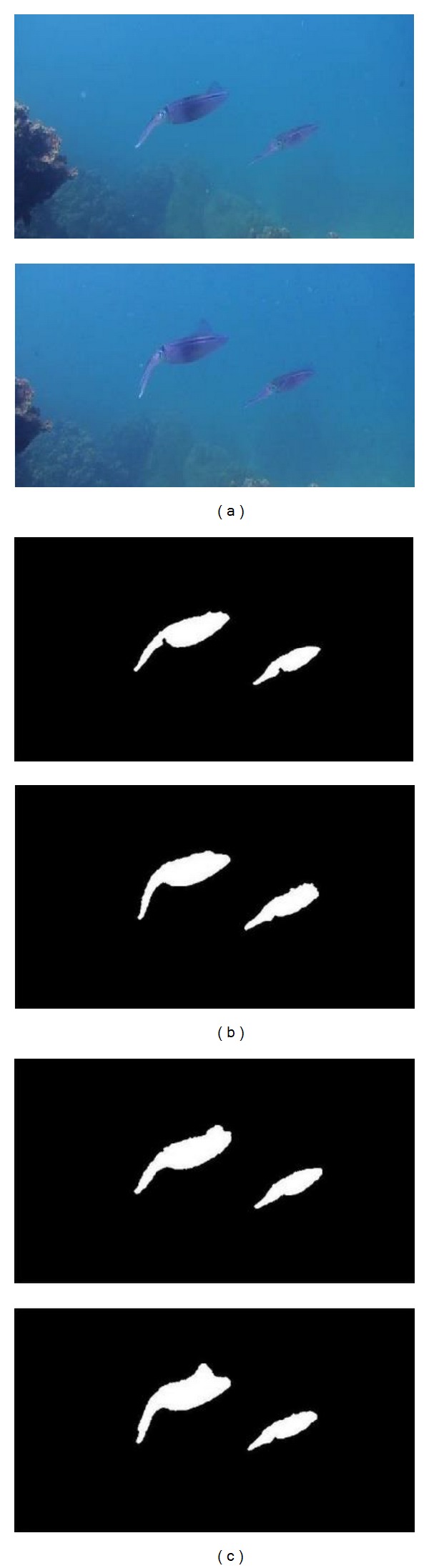
Results of multiple moving object detection. (a) Original images. (b) Gaussian background modeling method. (c) The proposed method.

In order to demonstrate the efficiency of the proposed method for detecting underwater moving object, the classic Gaussian background modeling method is selected as the reference which is used to compare it with our proposed method. The detection results are shown in Figures 3, 4, and 5.

According to the detection results, the Gaussian background modeling method has the ability to roughly detect contours of object. However the detected contours are not complete, especially for those parts which are similar to the background. In contrast to the results given by the Gaussian method, the contours of objects given by the proposed hierarchical method are more complete. The detected results are more precise. The criteria *C*_good_ and *C*_false_ [19] are employed to achieve quantized evaluation, as

(20) $$\begin{array}{c} C_{\text{good}}=\frac{\text{card}\left\{\Omega_{\text{in}}\cap \Omega_{\text{o}}\right\}}{\text{card}\left\{\Omega_{\text{o}}\right\}},\\ C_{\text{false}}=\frac{\text{card}\left\{\Omega_{\text{in}}\cap \Omega_{\text{b}}\right\}}{\text{card}\left\{\Omega_{\text{b}}\right\}}, \end{array}$$

where Ω_in_ is the detected object region, Ω_o_ is the real object region, and Ω_b_ is the background region. *C*_good_ denotes the ratio of the detected region to the real object region and *C*_false_ is the ratio of the false detected region to the background region. The performance evaluation is shown in Table 1.

From the results shown in Table 1, for the underwater moving object detection, the proposed hierarchical method, has better performance in contrast to the Gaussian background modeling method. More precise results can be obtained by our method. The mean value of *C*_good_ is increased to 0.9792 and the mean value of *C*_false_ is decreased to 0.0094. For Figure 3 and Figure 5, *C*_good_ obtained by the proposed method is very close to 1 while *C*_false_ is very small. It is indicated that the detected region by the proposed method can generally cover the real object and there is a very little background included in the results. For Figure 4 *C*_good_ achieved is relatively lower than that for Figure 3 and *C*_false_ is the lowest one among all results. Overall it is demonstrated that our method is feasible, effective, and sufficiently accurate for the underwater moving object detection.

## 5. Conclusion

Inspired by the frog visual mechanism, the frog visual information processing mode is simulated to establish a bionic underwater object detecting method. By using the illumination information of the input image a hierarchical background model is established to detect underwater moving objects. The experimental results demonstrate that the proposed method detects underwater moving objects effectively and accurately. In this paper the visual mechanism in the visual system of frogs is modeled preliminarily and further research work will focus on this field to achieve a more complete bionic model.

## Figures and Tables

**Algorithm 1:**
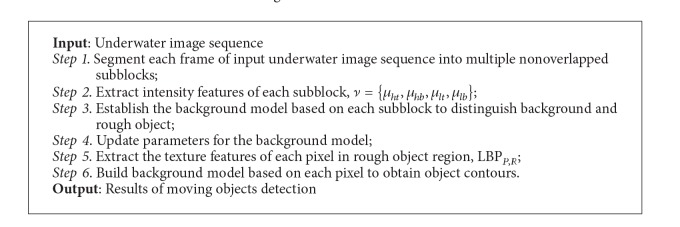
Hierarchical background modeling algorithm.

**Table 1:** Performance comparison.

	Figure 3		Figure 4		Figure 5		Mean value	
	*C*_good_	*C*_false_	*C*_good_	*C*_false_	*C*_good_	*C*_false_	*C*_good_	*C*_false_
Gaussian background modeling method	0.9713	0.0183	0.8660	0.0091	0.9657	0.0213	0.9343	0.0162
Hierarchical background modeling method	0.9906	0.0092	0.9589	0.0082	0.9881	0.0107	0.9792	0.0094

## References

[1] Zodiatis D, Lardner R, Solovyov D (2011). Predictions for oil slicks detected from satellite images using My Ocean forecasting data. *Ocean Science Discussions*.

[2] Corchs S, Schettini R (2010). Underwater image processing: State of the art of restoration and image enhancement methods. *EURASIP Journal on Advances in Signal Processing*.

[3] Wang HB, Dong X, Shen J, Wu XW, Chen Z (2013). Saliency-based adaptive object extraction for color underwater images. *Applied Mechanics and Materials*.

[4] Wang X, Lv G, Xu L (2012). Infrared dim target detection based on visual attention. *Infrared Physics and Technology*.

[5] Huang FC, Li M, Shi AY, Tang M, Xu L (2011). Insect visual system inspired small target detection for multi-spectral remotely sensed images. *Journal of China Institute of Communications*.

[6] Chen S, Zheng Y, Cattani C, Wang W (2012). Modeling of biological intelligence for SCM system optimization. *Computational and Mathematical Methods in Medicine*.

[7] Barat C, Rendas MJ A robust visual attention system for detecting manufactured objects in underwater video.

[8] Walther D, Edgington DR, Koch C Detection and tracking of objects in underwater video.

[9] Wang XF, Wang HY, Wang S (2011). Underwater object detection based on background subtraction and a saliency map. *Journal of Shangdong University (Engineering Science)*.

[10] Wang Z, Chen Z, Xu X, Wu L (2009). A fuzzy region understanding tactic for object tracking based on frog’s vision characteristic. *Acta Automatica Sinica*.

[11] Lettvin JY, Maturana HR, McCulloch WS (1959). What the frog’s eye tells the frog’s brain. *Proceedings of the IRE*.

[12] Yang W, Tang YY, Fang B, Shang Z, Lin Y (2013). Visual saliency detection with center shift. *Neurocomputing*.

[13] Zhao L, Wang TZ, Liu YH (2003). Research on frog visual behavior and its computer simulation. *Journal of Wuhan University of Tecnology (Information Management Engineering)*.

[14] Heikkilä M, Pietikäinen M (2006). A texture-based method for modeling the background and detecting moving objects. *IEEE Transactions on Pattern Analysis and Machine Intelligence*.

[15] Chen Z, Chen X, He H (2007). Moving object detection based on improved mixture Gaussian models. *Journal of Image and Graphics*.

[16] Guo Z, Zhang D, Mou X Hierarchical multiscale LBP for face and palmprint recognition.

[17] Zheng Y, Song Q, Chen S (2013). Multiobjective fireworks optimization for variable-rate fertilization in oil crop production. *Applied Soft Computing Journal*.

[18] Zheng Y, Chen S (2013). Cooperative particle swarm optimization for multiobjective transportation planning. *Applied Intelligence*.

[19] Sun Y, Fisher R (2003). Object-based visual attention for computer vision. *Artificial Intelligence*.

